# Genome-Wide Identification and Expression Pattern Analysis of the *HAK/KUP/KT* Gene Family of Cotton in Fiber Development and Under Stresses

**DOI:** 10.3389/fgene.2020.566469

**Published:** 2020-11-19

**Authors:** Xu Yang, Jingjing Zhang, Aimin Wu, Hengling Wei, Xiaokang Fu, Miaomiao Tian, Liang Ma, Jianhua Lu, Hantao Wang, Shuxun Yu

**Affiliations:** ^1^School of Agronomy Sciences, Zhengzhou University, Zhengzhou, China; ^2^State Key Laboratory of Cotton Biology, Institute of Cotton Research of CAAS, Anyang, China

**Keywords:** HAK/KUP/KT, cotton, expression patterns, fiber development, stress

## Abstract

The potassium transporter family HAK/KUP/KT is a large group of proteins that are important in plant potassium transport and plays a crucial role in plant growth and development, especially in economic crops. Although *HAK/KUP/KT* genes have been identified in many species, research on these genes in cotton is still quite rare. In this study, in total, 21, 24, 45, and 44 *HAK/KUP/KT* genes were identified in *Gossypium arboreum*, *Gossypium raimondii*, *Gossypium hirsutum*, and *Gossypium barbadense*, respectively. Phylogenetic analysis showed that these genes were divided into four clusters. The *G. hirsutum* gene promoters contained diverse *cis*-regulatory elements, such as drought-responsive elements, low temperature-responsive elements, and other elements. The RNA-seq data and qRT-PCR results showed that *HAK/KUP/KT* genes had different expression patterns in fiber development. The qRT-PCR results of drought and NaCl treatment indicated that *HAK/KUP/KT* genes might play important roles in abiotic stress responses. These results will provide molecular insights into potassium transporter research in cotton.

## Introduction

Potassium is one of the essential nutrients for plant growth and development, and potassium is widely distributed in various tissues as the second most abundant cation in plants. Potassium plays an important role in maintaining the cytosolic pH, cell turgor, cell elongation, enzyme activation, osmotic adjustment, some physiological processes and radiocesium toxicity reduction in soil (Cherel et al., 2014; Cherel and Gaillard, 2019; Ashraf et al., 2019). Plants have evolved to adjust their K absorption and utilization efficiency through a series of mechanisms to modulate their tolerance to biotic and abiotic stresses (Cherel et al., 2014). For K^+^ acquisition and distribution, plants have two K^+^ transport systems: K^+^ channels and K^+^ transporters. K^+^ channels allow plants to absorb K^+^ via a low-affinity K^+^ uptake mechanism at high [K^+^]_ext_ (external K^+^ concentrations above 0.3 mM), while the K^+^ transporter is a high-affinity K^+^ uptake mechanism acting at low [K^+^]_ext_ (below 0.2 mM) (Wang and Wu, 2013). Therefore, potassium transporters play an important role in K^+^ transport for plants undergoing K^+^ deficiency.

Potassium transporters can be divided into four families: the KT (K^+^ transporter)/HAK (high-affinity K^+^)/KUP (K^+^ uptake) family, Trk/Ktr/HKT family, KEA (K^+^ efflux antiporter) family, and CHX (cation/hydrogen exchanger) family (Gupta et al., 2008). The HAK/KUP/KT family contains high-affinity K^+^ transport carriers and is mainly responsible for the absorption and transport of K^+^ by plants. HAK/KUP/KT was first found in bacteria (Epstein and Kim, 1971), while plant HAK/KUP/KT, which is the largest K^+^ transporter family in plants, was first isolated from barley (*HAK1*) and *Arabidopsis* (*KUP1*/*KT1* and *KUP2*/*KT2*) based on homology to fungal HAK and bacterial KUP (Santa-María et al., 1997; Rehman et al., 2017). The plant HAK/KUP/KT transporter family has been described as electrochemical potential-driven transporters, and they are likely K^+^–H^+^ symporters (Santa-María et al., 1997). The KT/HAK/KUP gene family has been identified in many species. For example, there are 13 members in *Arabidopsis*, 27 members in rice, 27 members in maize, 16 members in peaches, 19 members in tomatoes, 56 members in wheat, 21 members in cassava, and 29 members in soybeans (Gupta et al., 2008; Zhang et al., 2012; Hyun et al., 2014; Scherzer et al., 2015; Song et al., 2015; Rehman et al., 2017; Cheng et al., 2018; Ou et al., 2018).

HAK/KUP/KT transporters have been found to play important roles in the regulation of plant growth and development, salt tolerance, and osmotic potential regulation (Wang and Wu, 2017; Li et al., 2018b). It has been discovered that *AtKUP5* is expressed only in root hairs and functions as a K^+^ flux sensor (Al-Younis et al., 2018). The mutation of *AtKUP7* led to a decrease in the K^+^ uptake rate and K^+^ content in xylem sap under low-K^+^ stress, so the mutant showed leaf chlorosis (Han et al., 2016). *OsHAK16* mediates K^+^ uptake and root-to-shoot transportation at a range of external K^+^ concentrations, thereby contributing to the maintenance of K homeostasis and salt tolerance in rice shoots (Feng et al., 2019). *OsHAK1* plays an essential role in K^+^-mediated rice growth and salt tolerance over low and high K^+^ concentration ranges (Chen et al., 2015).

Cotton is a crucial economic crop worldwide, and cotton fiber plays an important role in the textile industry. Potassium affects not only the yield of cotton but also the fiber quality (Hamamoto et al., 2015; Yang et al., 2016b). In developing fibers, the cell turgor is largely influenced by an influx of water driven by primary osmotica, wherein soluble sugars, K^+^, and malate account for 80% of the total osmotic potential (OP) (Ruan et al., 1997; Smart et al., 1998). K^+^ could contribute to an increase in cell turgor pressure during fiber elongation, thus participating in fiber development (Yang et al., 2016). *GhKT2*, which is a potassium transporter gene, has been identified in cotton. *Arabidopsis* lines overexpressing *GhKT2* were larger and showed greater K^+^ accumulation compared to the wild type (WT), and the net K^+^ influx rate of *GhKT2*-transgenic *Arabidopsis* lines in the root meristem zone was significantly greater than that of the WT (Wang et al., 2018). These findings showed that HAK/KUP/KT members might play an important role in cotton fiber development and responses to various stresses.

Here, we identified 134 HAK/KUP/KT family genes from the genomes of *G. arboreum*, *G. raimondii*, *G. hirsutum*, and *G. barbadense*. We examined their distributions in the genome, the characteristics of the motif distributions and gene structures, and the cis-elements in promoter regions and expression profiles. These results provide a foundation for the further functional characterization of potassium transporters in cotton.

## Materials and Methods

### Plant Materials and Treatments

Upland cotton TM-1 was used for tissue quantitative real-time RT-PCR analysis, and the plants were grown at Anyang (AY), Henan, China. The fiber samples were separated from the ovules 5, 10, 15, 20, and 25 days postanthesis (DPA) for RNA extraction. For RNA extraction, 0 and 3 DPA ovules were used directly.

TM-1 seeds were grown in hydroponic boxes containing Hoagland’s solution for stress treatments in a greenhouse with a photoperiod of 16 h light at 28°C/8 h at 22°C dark until the seedlings reached the three-leaf stage. Roots at the three-leaf stage of seedling development were then subjected to K^+^ deficiency (0.03 mM K^+^) treatment, salt stress (300 mM NaCl), and dehydration (18% PEG6000). Leaves were collected at 0 h, 3 h, 6 h, 9 h, 12 h, 24 h, and 48 h after the stress treatments began. All plant samples were collected with three biological replicates and immediately frozen in liquid nitrogen. All samples were then stored at -80°C until RNA isolation.

### Identification of Potassium Transporters in Cotton Species

Genome and protein sequence data for four cotton species (*G. arboreum*, *G. raimondii*, *G. hirsutum* and *G. barbadense*) were downloaded from CottonFGD^1^, and *Arabidopsis thaliana* data were downloaded from NCBI^2^. Potassium transporter proteins in *G. hirsutum* were predicted using the hidden Markov model (HMM) profile obtained from Pfam^3^ with e < 1e-100 and score > 300. To obtain all the potassium transporters in *G. hirsutum*, *Arabidopsis thaliana* potassium transporters were used as query sequences to search using Blastp, with an evaluation cutoff of 1e-10. Then, the results of HMM and Blastp were merged to select the common genes as potassium transporter candidate genes in *G. arboreum*, *G. raimondii*, *G. hirsutum* and *G. barbadense*. Furthermore, the online Simple Modular Architecture Research Tool (SMART) was used to confirm the conserved domain for all the candidate POT protein sequences (Letunic et al., 2015).

### Phylogenetic Analysis of HAK/KUP/KT Proteins

The amino acid sequences of the *HAK/KUP/KT* genes of *A. thaliana, O. sativa, G. arboreum, G. raimondii, G. hirsutum*, and *G. barbadense* were saved as FASTA format files, and multiple sequence alignments of *HAK/KUP/KT* genes were performed by Clustal X 2.0 (Thompson et al., 2003) using the Molecular Evolutionary Genetics Analysis (MEGA) version 6.0 program (Tamura et al., 2013). A phylogenetic tree was constructed using MEGA 6.0 by the neighbor-joining method. Support for the tree topology was evaluated by using a bootstrap analysis with 1000 replicates. The online tool iTOL^4^ was used to modify the phylogenetic tree (Letunic and Bork, 2016).

### Analysis of Motifs and Gene Structures

The gene structure data of HAK/KUP/KTs were extracted from the genome annotation files. The Multiple Expectation Maximization for Motif Elicitation (MEME v4.9.1) utility program was used to analyze the conserved motifs among GhPOT proteins. Gene Structure Display Server (GSDS) 2.0^5^ was used to represent the gene structures on the basis of the coding sequences, untranslated regions, and intron and exon data from the gene annotation file.

### Gene Locations of the *HAK/KUP/KT* Gene Family

The locations of HAK/KUP/KTs on the chromosomes were obtained from the gene annotation (GFF) files and displayed by Mapchart (Voorrips, 2002).

### Analysis of *cis*-Regulatory Elements

For the identification of cis-regulatory elements in *HAK/KUP/KT* genes, a 2000-bp region upstream of each translation start site was acquired by aligning the coding sequences with the genomic sequences. Regulatory elements were then predicted using the PlantCARE database^6^.

### Gene Expression and qRT-PCR Analysis of GhPOT Genes

Raw RNA-seq data were downloaded from the NCBI Sequence Read Archive (SRA: PRJNA248163). TopHat2 (Kim et al., 2013) and cufflinks (Trapnell et al., 2010) were used to analyze the RNA-seq expression, and the gene expression was measured in fragments per kilobase million (FPKM). The TBtools program (Chen et al., 2018) was used to display the heatmap of gene expressions. The 2^–ΔΔCT^ method was used to calculate the relative expression levels of the GhPOTs (Livak and Schmittgen, 2001).

The total RNA was extracted using TIANGEN (TransGen Biotech, Beijing, China) according to the manufacturer’s instructions. cDNA was synthesized using the PrimeScript^TM^ RT Reagent Kit with gDNA Eraser (Takara, Dalian, China). GoTaq^®^ qPCR Master Mix (Promega, Beijing, China) was used for qPCR amplification in QuantStudio^TM^ 5 (Thermo Fisher Scientific, Shanghai, China). The qRT-PCR conditions were as follows: 95°C for 5 min, followed by 40 cycles of 95°C for 15 s and 61°C for 1 min, with a final extension at 72°C for 5 min. qRT-PCR (Promega, Madison, WI, United States) was performed on an ABI 7500 Real-Time PCR system (Applied Biosystems, United States) with three technical and biological replicates. The standard deviation was used to calculate the qRT-PCR results error. All qRT-PCR primers are listed in Supplementary Table S1.

## Results

### Identification of Potassium Transporters in Four Cotton Species

Combining the results of HMM and Blastp, a total of 21, 24, 45, and 44 HAK/KUP/KT genes were identified in *G. arboreum*, *G. raimondii*, *G. hirsutum*, and *G. barbadense*, respectively. All the predicted HAK/KUP/KT proteins have a typical “k_trans” domain, which is a symbol of HAK/KUP/KT potassium transporter family members. The names of all the HAK/KUP/KTs were determined by their protein name annotations in CottonFGD^7^ and their location order on the chromosomes. The lengths of the HAK/KUP/KT protein sequences ranged from 719 aa (GaPOT5-2) to 939 aa (GaPOT4-2); 619 aa (GrPOT4-1) to 858 aa (GrPOT7-1); 494 aa (GhHAK13-1) to 858 aa (GhPOT7-1, GhPOT7-3); and 584 aa (GbPOT6-4) to 858 aa (GbPOT7-1). The molecular weights, pI values, GRAVY scores and subcellular localizations of the *HAK/KUP/KT* genes are shown in Supplementary Table S2.

### Phylogenetic Analysis of HAK/KUP/KT Proteins

**FIGURE 1:**
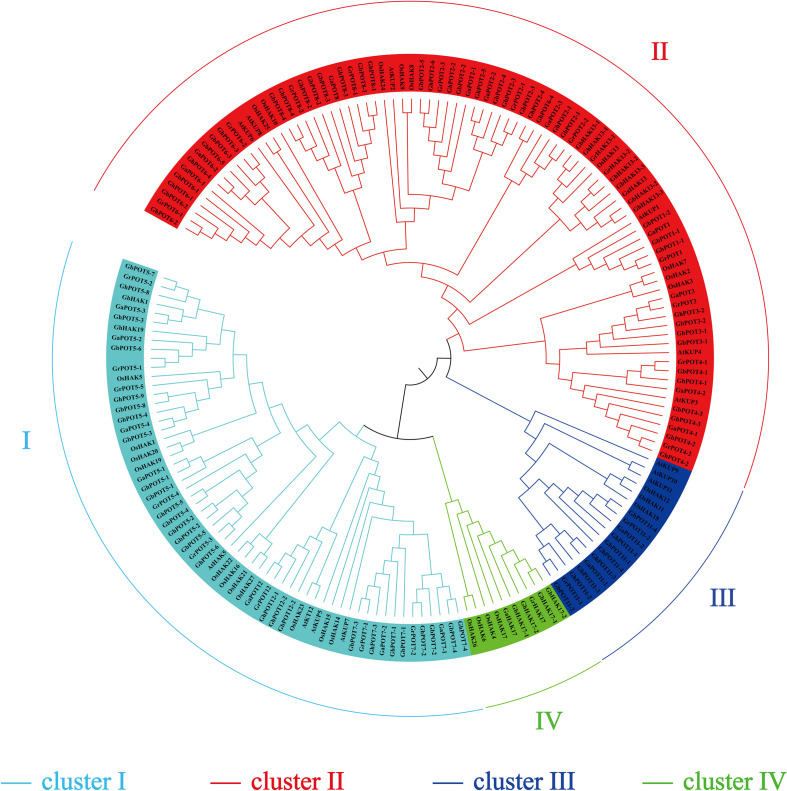
Phylogenetic tree of HAK/KUP/KT proteins. The predicted sequences from *O. sativa* (27), *A. thaliana* (13), *G. raimondii* (24), *G. arboretum* (21), *G. hirsutum* (45), and *G. barbadense* (44) were aligned with ClustalX 2.0, and MEGA 6.0 was used to construct the phylogenetic tree by the neighbor-joining (NJ) method.

A phylogenetic tree was constructed to examine the phylogenetic relationships between the HAK/KUP/KT protein sequences of cotton and other species. One hundred and seventy-four protein sequences were used to estimate the phylogenetic tree, including 45 from *G. hirsutum*, 21 from *G. arboreum*, 44 from *G. barbadense*, 24 from *G. raimondii*, 13 from *Arabidopsis*, and 27 from *O. sativa*. All the family members were divided into four clusters (clusters I, II, III, and IV) according to the classification criteria used for *Arabidopsis* and *O. sativa*. As shown in Figure 1, cluster II was the most abundant, including 23 GhPOTs, 11 GaPOTs, 24 GbPOTs and 13 GrPOTs and accounting for nearly 40.8% of all the *HAK/KUP/KT* genes. Cluster I accounted for 25.9%, including 16 GhPOTs, 7 GaPOTs, 14 GbPOTs and 8 GrPOTs. Cluster I accounted for 21.3%, with 10 GhPOTs, 4 GaPOTs, 9 GbPOTs and 5 GrPOTs. Cluster III had the smallest number, with only 6 cotton HAK/KUP/KTs.

### Analysis of Motifs and Gene Structures

**FIGURE 2:**
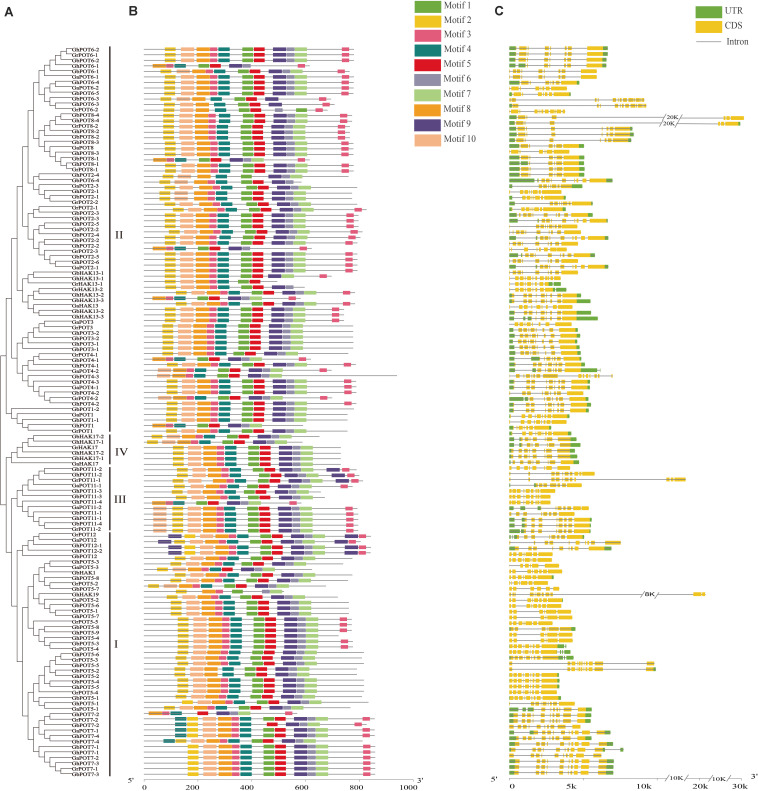
Phylogenetic relationships, motif analysis and exon-intron structures of GrPOTs, GaPOTs, GhPOTs, and GbPOTs. **(A)** Phylogenetic analysis of 134 HAK/KUP/KT proteins using MEGA 6.0 by the neighbor-joining (NJ) method. I, II, III, and IV were the clusters determined by the phylogenetic analysis. **(B)** The 10 motifs of 134 HAK/KUP/KT proteins were determined by MEME. Differently colored boxes represent different motifs. **(C)** Exon–intron structures of 134 *HAK/KUP/KT* genes. UTRs (untranslated regions), exons, and introns are indicated by green boxes, yellow boxes, and black lines, respectively.

A total of 10 motifs was identified by the online program MEME and named motifs 1-10. As shown in Figure 2, almost all the HAK/KUP/KTs contained all the motifs. Only 8 genes (GhPOT6-1, GbPOT8-1, GhPOT2-2, GhPOT1-1, GhHK13-3, GrPOT4-1, GhPOT11-4 and GhPOT7-2) did not contain motif 2, which was found at the beginning of every other sequence. Most genes had two copies of motif 3, except POT3, POT1, POT17, some POT2s, and most POT5s. Five POT7s contained two copies of motif 4 (GbPOT7-4, GhPOT7-4, GaPOT7-1, GbPOT7-2, and GrPOT7-2). Motif 9 was repeated twice in GbPOT11-2, GhPOT11-2, and GrPOT11-1 and three times in GrPOT12, GaPOT12, GhPOT12-1, and GhPOT12-2.

The gene structure was analyzed by GSDS. The results showed that the number of exons per gene ranged from 6 to 15 (Figure 2), but only *GrPOT6-2*, *GhPOT6-3*, *GbHAK13-1*, *GbPOT2-1*, and *GaPOT4-2* contained more than 11 exons. The structures of the genes in different clusters demonstrated different functions, but genes in the same cluster also showed different structures. Almost all the POT6 and POT8 genes in cluster II had 7 or 8 exons, and most other genes in cluster II contained 9 exons. All of the POT7 genes in cluster I had 10 exons, but the other genes in cluster I had 8 or 9 exons.

### Chromosome Locations of the *HAK/KUP/KT* Gene Family in Four Cotton Species

**FIGURE 3:**
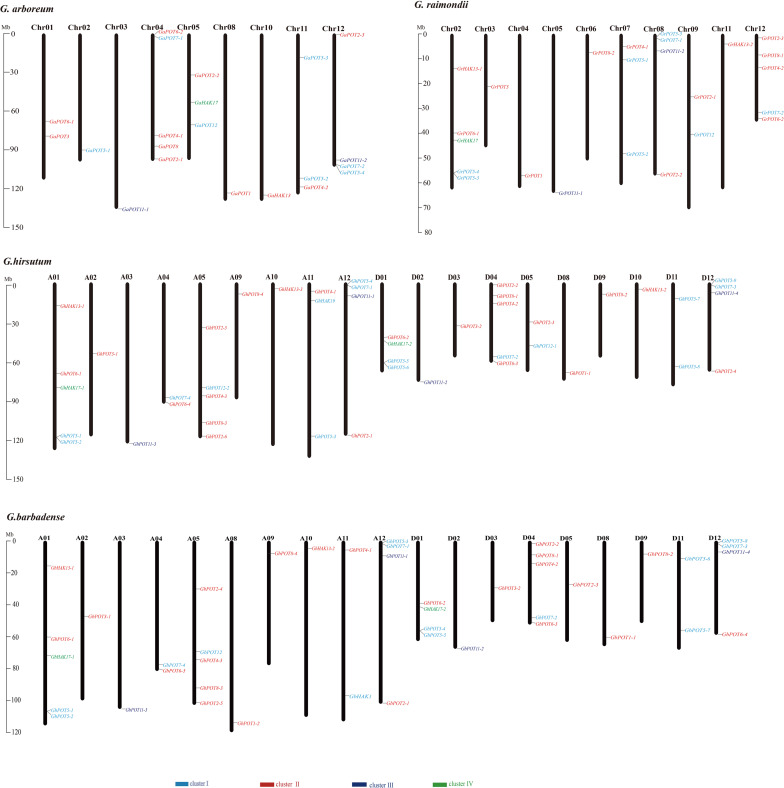
Chromosomal distributions of HAK/KUP/KT genes in *G. arboreum*, *G. raimondii*, *G. hirsutum*, and *G. barbadense*. The chromosome number is indicated above each vertical bar representing one chromosome. The gene names are indicated on the right side. The HAK/KUP/KTs in each cluster are shown in the same color. The lengths of the chromosomes are shown in Mb (millions of bases).

The *HAK/KUP/KT* genes were mapped to the chromosomes of the four cotton reference genomes (Figure 3). All the genes were unevenly distributed on the chromosomes, except GbHAK13-3, which was located on Scaffold3708. The results indicated that 23 GhPOTs and GbPOTs were located on At subgenome chromosomes, while 22 GhPOTs and 20 GbPOTs were located on Dt subgenome chromosomes. However, no GhPOTs were located on A06, A07, A08, D06, or D07. *G. arboreum* had three chromosomes with no *HAK/KUP/KT* genes (Chr06, Chr07, and Chr09), and *G. raimondii* had two chromosomes with no *HAK/KUP/KT* genes (Chr01 and Chr10). Comparing the gene distributions and gene numbers among the four cotton species showed that the chromosome distributions and gene numbers were conserved in the evolution of cotton from diploid to allotetraploid.

### Analysis of Cis-Regulatory Elements in Upland Cotton

**FIGURE 4:**
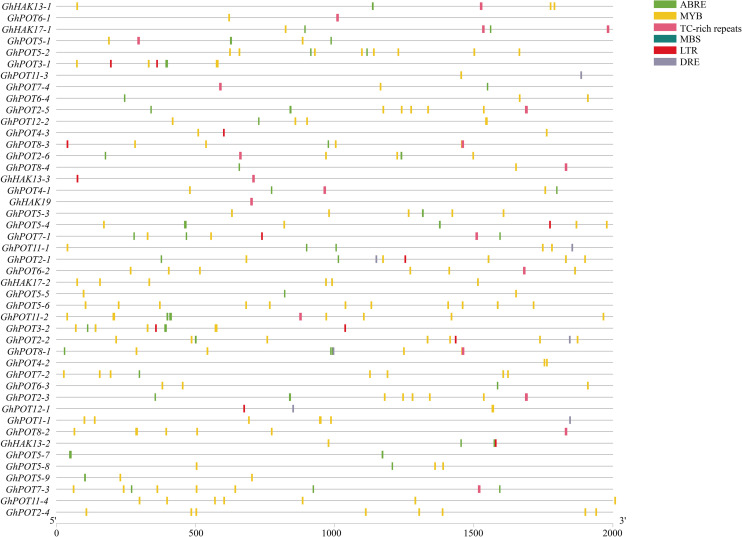
Distribution of major stress-related *cis*-elements in the promoter regions of GhPOTs. The locations of these *cis*-elements were confirmed using the PlantCARE database. Different *cis*-elements are represented by boxes of different colors.

To analyze the possible factors influencing the expression patterns of GhPOT gene family members, the 2000-bp sequences upstream of the transcription initiation sites of cotton *HAK/KUP/KT* genes were used for the prediction of cis-regulatory elements (Figure 4). The main stress elements were STRE, ABRE (ABA), MYB, TC-rich repeats (defense and stress response), MBS (drought), LTR (low-temperature response), and DRE. Notably, ABRE, MYB and DRE are all drought-relative responsive elements. The promoters of some *GhPOTs*, such as *GhPOT5-2* (2 ABRE, 1 MBS, and 8 MYB), *GhPOT5-6* (1 MBS and 11 MYB), and *GhPOT2-2* (2 ABRE, 1 DRE, 2 MBS, and 7 MYB), contained many drought-responsive elements. In addition, the *GhPOTs* contained other specific *cis*-regulatory elements (Supplementary Table S3), for example, cell cycle regulation (*GhPOT4-1*), seed-specific regulation (*GhPOT11-3*, *GhPOT5-3*, *GhPOT1-1* and *GhPOT5-9*), meristem expression (*GhPOT11-3*, *GhPOT2-5*, *GhPOT2-6*, *GhPOT11-1*, *GhPOT2-1*, *GhPOT6-2*, *GhPOT5-6*, *GhPOT2-2*, *GhPOT2-3* and *GhPOT2-4*) and circadian control (*GhPOT2-6*, *GhPOT8-4*, *GhPOT5-4*, *GhPOT11-1*, *GhPOT5-6*, *GhPOT2-2*, *GhPOT4-2*, *GhPOT6-3*, *GhPOT12-1*, *GhPOT8-2* and *GhPOT5-9*). Figure 4 and Supplementary Table S4 show that the ABRE and MBS elements were enriched in the proximal region of the promoter from TSS, and DRE and TC-rich were enriched in the distal region of the promoter from TSS.

### Gene Expression Analysis of GhPOT Genes

**FIGURE 5:**
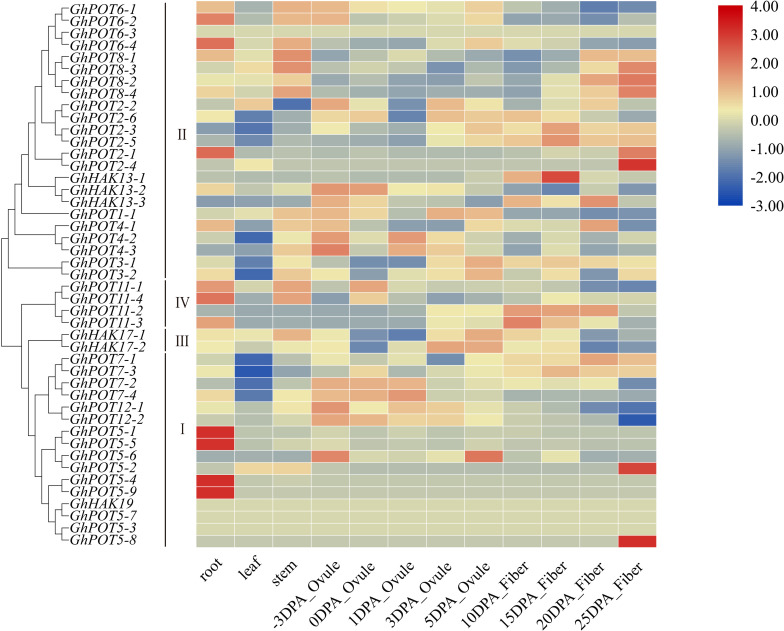
Transcriptome data of GhPOTs in different tissues. DPA indicates days postanthesis. Gene names are shown on the left. The scale bar on the right shows the colors used to represent the log2-transformed FPKM values for each gene.

Publicly available RNA-seq databases were used to analyze the expression patterns of GhPOT genes in cotton roots, leaves, stems, ovules from -3 DPA (days postanthesis) to 5 DPA and fibers from 10 DPA to 25 DPA. The genes in cluster I showed significantly specific expression in different tissues. *GhPOT5-1*, *GhPOT5-5*, *GhPOT5-4*, and *GhPOT5-9* were highly expressed in roots, while almost no expression was observed in other tissues. *GhPOT5-2* and *GhPOT5-8* were highly expressed in 25 DPA fibers and had no expression in other fiber development stages and tissues. Most GhPOTs in clusters II, III and IV had different expression levels in different tissues. Figure 5 shows that most of the genes in clusters II and III were highly expressed in different fiber development stages. The 6 *GhPOT2* genes and 4 *GhPOT8* genes in cluster II were highly expressed in 20∼25 DPA fibers, which correspond to secondary cell wall biosynthesis. Two *GhPOT7* genes in cluster I (*GhPOT7-2* and *GhPOT7-4*) and 2 *GhPOT12* genes in cluster III were highly expressed in the -3∼1 DPA samples. *GhPOT7-1* and *GhPOT7-3* were highly expressed in fibers from 5 to 20 DPA, and *GhPOT11-2* and *GhPOT11-3* were specifically expressed in fibers from 10 to 20 DPA.

**FIGURE 6:**
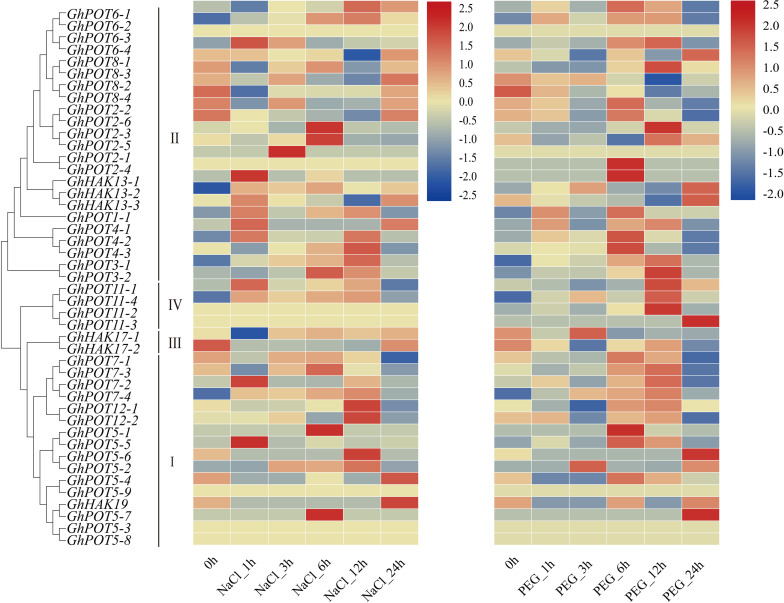
Transcriptome data of GhPOTs in response to NaCl and PEG stresses. The treatments are shown at the bottom, and the genes and phylogenetic relationships are shown on the left. The scale bar on the right shows the colors used to represent the log2-transformed FPKM values for each gene.

During 24 h of drought and salt stress, most genes showed different expression levels and expression patterns (Figure 6), but some genes in the same subfamilies showed the same expression patterns. *GhPOT6* and *GhPOT4* were highly expressed after 6 h PEG treatment. *GhPOT3*, *GhPOT11*, and *GhPOT7* were highly expressed after 12 h PEG treatment. After 12 h NaCl treatment, the expressions of 7 genes increased to a maximum, and the expressions of 6 genes decreased to a minimum. From the transcriptome analysis, almost all the *HAK/KUP/KT* genes were upregulated, while *GhHAK17-2* was downregulated after salt stress.

### qRT-PCR Expression Analysis in Different Fiber Development Stages

**FIGURE 7:**
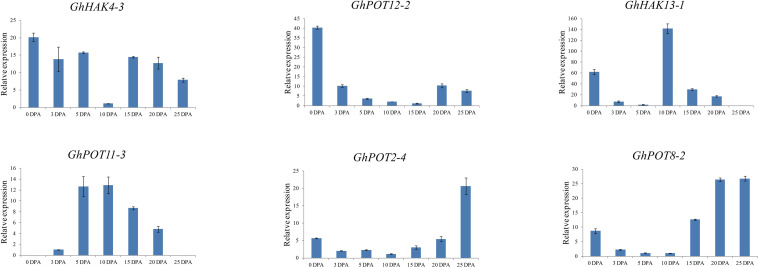
Expressions of GhPOT genes in different fiber development stages. The expressions of the GhPOT genes were determined by qRT-PCR using the total RNA isolated from TM-1 fiber. The error bars indicate the standard error (SE) of three biological replicates. DPA (days post anthesis) is the sampling period.

To investigate the possible functions of HAK/KUP/KT in fiber development, we selected 6 genes, which showed high expression levels in different fiber development stages in the transcriptome data (Figure 5) to examine their expression patterns. The results (Figure 7) showed that different genes had different expression patterns. The expression level of *GhHAK4-3* was high in each period of fiber synthesis (especially 0 DPA ovule), except in 10 DPA fiber. *GhPOT12-2* was significantly expressed in 0 DPA ovules, which is the initiation stage in fiber development. *GhPOT11-3* and *GhHAK13-1* were highly expressed in 5 and 10 DPA fibers, which are the expansion stages in fiber development. *GhPOT2-4* and *GhPOT8-2* were highly expressed in 20 and 25 DPA fibers, which are the secondary wall synthesis stages in fiber development.

### qRT-PCR Expression Analysis in Response to Multiple Stress Treatments

According to the analysis of cis-elements in promoter regions and previous studies of HAK/KUP/KTs in other plants, GhPOTs might be involved in stress responses. To verify this hypothesis, 12 GhPOTs were selected for qRT-PCR under K^+^ defiency, salt and drought treatments. The results revealed that all 12 GhPOTs could be induced by all three stresses to different degrees.

**FIGURE 8:**
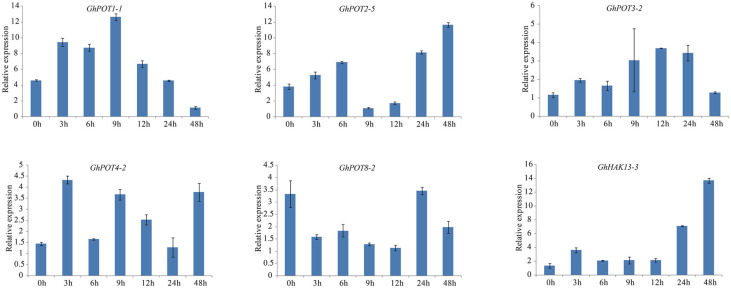
Expressions of GhPOT genes in response to potassium deficiency stress (0.03 mM KCl). The expressions of the GhPOT genes were determined by qRT-PCR using the total RNA isolated from TM-1 leaves at different time points (0, 3, 6, 9, 12, 24 and 48 h) of dehydration stress. The error bars indicate the standard error (SE) of three biological replicates.

As shown in Figures 8, 6, random selected genes were examined under potassium deficiency stress, and all showed basically the same expression pattern—after potassium deficiency treatment, the expression was upregulated for a few hours and then downregulated. *GhPOT1-1* and *GhPOT3-2* precisely followed this pattern, arriving at their peaks at 9 h and 12 h, respectively. *GhHAK13-3* and *GhPOT2-5*, showed upregulated expressions after the basic pattern and reached their maximum expression levels at 48 h. Although *GhPOT8-2* was downregulated after treatment, the maximum value was reached at 24 h after treatment, and its change trend followed the pattern.

**FIGURE 9:**
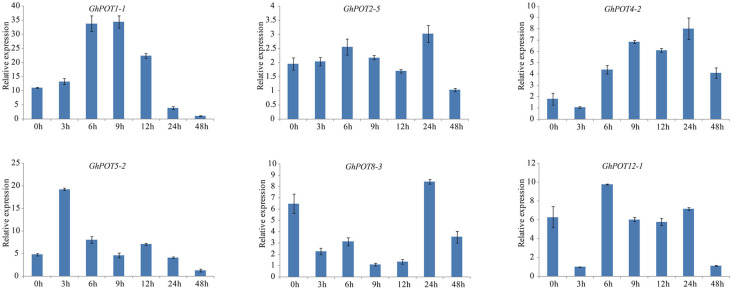
Expressions of GhPOT genes in response to dehydration stress (18% PEG6000). The expressions of GhPOT genes were determined by qRT-PCR using the total RNA isolated from TM-1 leaves at different time points (0, 3, 6, 9, 12, 24 and 48 h) of salt stress. The error bars indicate the standard error (SE) of three biological replicates.

Additionally, 6 genes that showed high expressions after PEG treatment according to transcriptome data were selected for the drought treatment. Under dehydration stress simulated by 18% PEG6000, all GhPOT genes were upregulated, albeit to different levels at different times (Figure 9). *GhPOT1-1*, *GhPOT4-2* and *GhPOT5-2* were all significantly upregulated. In particular, *GhPOT1-1* and *GhPOT5-2* responded rapidly to dehydration, and their expressions reached a maximum after 3 h of stress. Comparatively, *GhPOT2-5*, *GhPOT8-3*, and *GhPOT12-1* were moderately upregulated. *GhPOT4-2*, *GhPOT8-3* and *GhPOT12-1* showed the same change trend—their expression levels were immediately downregulated at 3 h after treatment and then oscillated.

**FIGURE 10:**
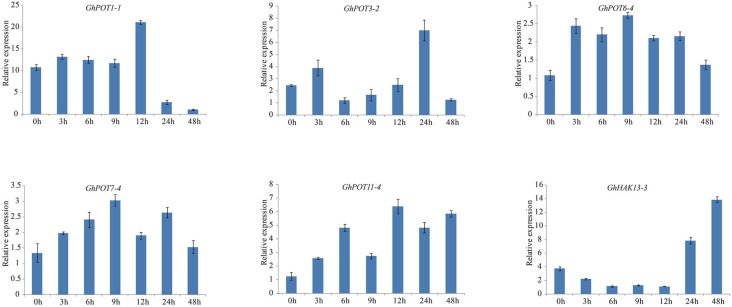
Expressions of GhPOT genes in response to salt stress (300 mM NaCl). The expressions of the GhPOT genes were determined by qRT-PCR using the total RNA isolated from TM-1 leaves at different time points (0, 3, 6, 9, 12, 24 and 48 h) of potassium deficiency stress. The error bars indicate the standard error (SE) of three biological replicates.

For the salt treatment, 6 genes that were highly expressed after NaCl treatment according to NaCl transcriptome data were used. After treatment with 300 mM NaCl, the expression levels of the 6 selected genes were all upregulated (Figure 10). However, *GhHAK13-3* was not upregulated initially. Instead, it was continuously downregulated until 12 h of treatment. The response of *GhHAK13-3* to stress was slower, and its upregulation became obvious after 24 h of stress. *GhPOT3-2*, *GhPOT6-4*, *GhPOT7-4*, and *GhPOT1-1* showed significantly reduced expression levels after 48 h of exposure to salt stress.

## Discussion

Potassium is one of the essential nutrients for plant growth and development, and potassium is widely distributed in various plant tissues as the second most abundant cation in plants. HAK/KUP/KT, as a potassium transporter family member, plays an important role in maintaining K + concentrations to affect the cytosolic pH, cell turgor, cell elongation, enzyme activation, and osmotic adjustment, as well as some physiological processes.

### Characterization of the *HAK/KUP/KT* Gene Family in Cotton

In this study, 45, 44, 21, and 24 potassium transporters were identified from the *G. hirsutum*, *G. barbadense*, *G. arboreum*, and *G. raimondii* genomes, respectively. The number of POT genes in *G. hirsutum* and *G. barbadense* was the sum of the numbers of POT genes in *G. arboreum* and *G. raimondii*. This is probably because *G. hirsutum* and *G. barbadense* are tetraploid plants that contain two genomes from their ancestors, *G. arboreum* and *G. raimondii*, and chromosome reduplication occurred. The *G. barbadense* and *G. hirsutum* chromosome locations were fundamentally consistent. The *G. hirsutum* At subgenome locations were similar to those of *G. raimondii*, and the Dt subgenome locations were similar to those of *G. arboreum*. The structures and locations of the genes showed evolutionary conservation. All the genes were classified into four clusters (I, II, III, and IV) based on their phylogenetic relationships. Cluster II was the largest cluster, not only in cotton but also in other species, representing 8 of 19 family members in tomatoes (Hyun et al., 2014), 9 of 27 in maize (Zhang et al., 2012), 12 of 21 in pears (Li et al., 2018), 11 of 21 in cassava (Ou et al., 2018) and 15 of 27 in rice (Gupta et al., 2008). Although HAK/KUP/KT transporters were reported to function in high-affinity K^+^ uptake in low-K^+^ environments (Ahn et al., 2004; Gierth et al., 2005), most cluster II transporters might instead play roles in low-affinity K^+^ transport (Gupta et al., 2008). *OsHAK7* and *OsHAK10*, belonging to cluster II, have been reported to mediate low-affinity K^+^ uptake in heterologous systems (Banuelos et al., 2002). It is possible that the cotton genes in Cluster II also participate in low-affinity K^+^ transport. Figure 2 shows that proteins in a group that share the same motifs are likely to share similar functions or have recent common evolutionary origins.

### HAK/KUP/KT Putative Functions in Fiber Development

Publicly available RNA-seq databases were used to analyze gene expressions. There is no doubt that potassium transporter genes are highly expressed in roots or stems because plants need roots and stems to transport or store K^+^ to complete biochemical reactions and maintain cell turgor. Figure 5 shows that the expression levels of HAK/KUP/KTs were completely different in different fiber development stages. *GhPOT4*, *GhPOT12* and *GhPOT7* were highly expressed in the initiation stage. *GhPOT2*, *GhPOT1*, *GhHAK17* and *GhPOT3* were highly expressed in the expansion stage, and *GhPOT8* and *GhPOT2* genes had high expressions in the secondary wall synthesis stage. Some genes even covered two or three stages. It has been reported that K^+^ could indirectly increase the maximal fiber elongation rate and final fiber length (Zhao et al., 2019). Two cotton varieties (Siza3 and Simian3) were treated with different K^+^ concentrations (K0, K1, and K2) in 2015 and 2016. The results showed that under K1 and K2 treatment, the fiber length of Siza3 increased by 3.9% and 8.24%, respectively, and the fiber length of Simian3 increased by 3.54% and 8.08%, respectively, compared with those at K0 (Zhao et al., 2019). In-depth studies have found that potassium deficiency can accelerate the synthesis of secondary wall cellulose and inhibit the fibers from obtaining carbohydrates from nearby leaves, thus disrupting the fiber development process and significantly reducing the fiber quality (Yang et al., 2016). The qRT-PCR (Figure 7) showed the same results as did the RNA-seq databases. *GhPOT12-1* was expressed in the initation stage. *GhPOT11-3* and *GhHAK13-1* were highly expressed in the expansion stage. *GhPOT11-3* and *GhHAK13-1* could possibly increase the K^+^ concentration to improve the fiber elongation rate. *GhPOT2-4* and *GhPOT8-2* were highly expressed in the secondary wall synthesis stage. Thus, we can infer that *GhPOT2-4* and *GhPOT8-2* reduce the K^+^ concentration to promote secondary wall cellulose synthesis and obtain carbohydrates from leaves.

It has been reported that the knockout of some *HAK/KUP/KT* genes in plants affects not only K^+^ acquisition and transportation but also the root and shoot morphology. The root hairs of *AtHAK5*-overexpressing plants were more developed, even under low-potassium conditions (Zhao et al., 2016). A triple mutation (*AtKUP2* and its homologs, *AtKUP6* and *AtKUP8*) causes an increase in the root cell size, suggesting that these transporters negatively regulate turgor-dependent shoot growth (Osakabe et al., 2013). The fiber cell, as a model single cell, may be induced by HAK/KUP/KT to develop its form and length. These results suggested that *HAK/KUP/KT* genes that are highly expressed in different fiber stages could influence fiber development by regulating the K^+^ concentration.

### Putative Functions of HAK/KUP/KT in Stress

Gene promoters specifically regulate transcription initiation and can control the expression levels of specific genes in cells. The level of gene expression is directly related to the gene function. Thus, promoter expression activity can effectively predict the functions of specific genes. A previous study demonstrated that many *HAK/KUP/KT* genes are involved in drought, salt, and osmotic stress responses and ionic homeostasis (Grabov, 2007; Wang and Wu, 2017; Li et al., 2018). In general, most GhPOTs (except genes with very low or no expression in the investigated tissues) were induced by salt and drought, even though the induction of some GhPOTs was slight.

### K^+^ Deficiency

Potassium is an important element for plant growth. Under low-K^+^ stress, plants can absorb K^+^ through HAK/KUP/KT to maintain a steady state in the cytoplasm, which represents a major mechanism of plant response to low-K^+^ stress (Li et al., 2018). *AtHAK5* and *AtKT1* are the two essential transporters mediating high-affinity Rb^+^ (K^+^) uptake in the roots of Arabidopsis, and the residual Rb^+^ (K^+^) concentration detected in double-mutant roots was insufficient to sustain plant growth (Pyo et al., 2010). *ZmHAK5* was characterized as a high-affinity K^+^ transporter in maize. It was found by experimental comparison that plants with a loss of *ZmHAK5* function exhibited defective K^+^ uptake under low-K^+^ conditions, whereas *ZmHAK5*-overexpressing plants showed increased K^+^ uptake activity and improved growth (Qin et al., 2019). As shown in Figure 8, almost all the tested genes were upregulated after K^+^ deficiency, especially *GhHAK13-3* and *GhPOT1-1*. Therefore, we may expect that HAK/KUP/KT genes could increase the potassium ion absorption capacity under K^+^-deficiency. Overall, HAK/KUP/KT genes enhance the K^+^ deficiency tolerance, which is important for current agricultural production.

### Salt Stress

Salt stress is an abiotic stress relevant to modern agricultural production. In this study, transcriptome analysis indicated that most GhPOT genes were responsive to salt stress, and the qRT-PCR results showed that all the selected genes were upregulated after treatment with high concentrations of NaCl. There have been some reports of HAK/KUP/KT genes in other species that could relieve salt stress in plants. Salt stress significantly decreased the root net K^+^ uptake rate in WT rice and almost completely blocked net K^+^ uptake in *Oshak1* mutants when the K^+^ concentration was below 0.05 mm. However, plants overexpressing *OsHAK1* were more tolerant of salt stress compared to the wild type. The same results were shown in *HvHAK1*, *LeHAK5*, and *CaHAK1* (Martínez-Cordero et al., 2004; Nieves-Cordones et al., 2007; Fulgenzi et al., 2008). *GhPOT5*, which is a homolog of *OsHAK1*, showed higher expression after treatment than did other genes (Figure 6). *AtHKT1* provides a key mechanism for protecting leaves from salt stress (Hamamoto et al., 2015), and *GhPOT1*, which is homologous to *AtHKT1*, showed significantly increased expression after salt treatment (Figures 6, 10).

### Drought Stress

**FIGURE 11:**
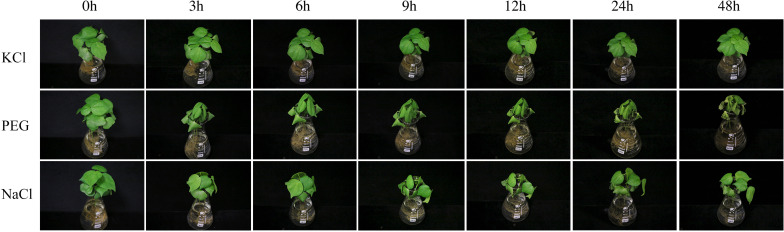
TM-1 phenotypic pictures at different time points (0, 3, 6, 9, 12, 24 and 48 h)after 0.03 mM KCl, 18%PEG and 300 mM NaCl treatments.

In the present study, the results of a *cis*-acting element analysis of the GhPOT promoter regions revealed many *cis*-acting elements that significantly responded to drought stress, such as ABRE, MYB, and MBS, and they were distributed in both proximal and distal regions of the promoter from TSS. ABREs are ABA-responsive elements that have been reported to play an important role in the drought stress response (Sah et al., 2016), and most GhPOT promoters possess ABA-responsive elements. Moreover, transcriptome analysis showed that most GhPOT genes were clearly upregulated after drought stress (Figure 6). During drought stress, root growth and the rate of K^+^ diffusion toward the roots in the soil are both restricted, thus limiting K^+^ acquisition (Wang et al., 2013). Plants that are exposed to drought stress over a long time can form reactive oxygen species (ROS), which enables the activation of high-affinity K^+^ uptake (Ashley et al., 2006). As shown in Figure 11, the plants showed significant wilt after the treatment 3h compared with other treatments. Here, most of the examined genes increased their expression after drought treatment (Figures 6, 9), especially *GhPOT5-2* immediately improve its expression after treatment 3h. The other five genes enhanced their expression level at 6h, especially *GhPOT1* (Figure 9). However, under continuous exposure to drought stress, high levels of ROS lead to leaf damage (Wang et al., 2013). All the selected genes reached their maximum expressions after 24 h of drought treatment, then began to decline (Figure 9). On the basis of this evidence and previous studies (Grabov, 2007; Wang et al., 2013; Li et al., 2018), the *HAK/KUP/KT* genes might improve drought tolerance.

Generally, GhPOT genes were extensively induced by multiple abiotic stresses, revealing that these genes perform important functions in response to drought, salt and K^+^ deficiency stresses in cotton. These results implied that the GhPOT gene family plays an important role in improving the cotton stress tolerance. Because potassium has similar chemical properties to those of cesium, three K^+^ transport genes were found in rice by yeast library screening, and it was found that the three genes had sensitive responses to Cs^+^-induced root growth inhibition, expecially *OsHAK5* (Yamaki et al., 2017). As expected, *GhPOT5* and *GhPOT1* could have significant responses to drought, salt and K^+^ deficiency stresses. Thus, *GhPOT5* and *GhPOT1* may have crucial effects on stress responses.

## Conclusion

In this study, we identified 45, 21, 44, and 24 *HAK/KUP/KT* genes in *G. hirsutum*, *G. arboreum*, *G. barbadense*, and *G. raimondii*, respectively. Phylogenetic analysis grouped these genes into four clusters. Chromosome location, conserved motif, conserved domain and gene structure analyses of all the cotton *HAK/KUP/KT* genes were subsequently performed. The *G. hirsutum* gene promoters contain diverse cis-regulatory elements. The RNA-seq data showed that *HAK/KUP/KT* genes are specifically expressed in different stages of fiber development. PEG and NaCl treatment transcriptomes indicated that *HAK/KUP/KT* genes may have disparate functions when facing drought and salt stress. Upon combining the qRT-PCR results, all the results indicate that *HAK/KUP/KT* genes might participate in stress responses and improve the stress resistance. In conclusion, these results will provide molecular insights into potassium transporter research in cotton. Meanwhile, some genes can be studied further to explore their functions, like *GhPOT2-4*, *GhPOT8-2*, *GhPOT1*, and *GhPOT5*,

## Data Availability Statement

All datasets generated for this study are included in the article/Supplementary Material.

## Author Contributions

HTW and XY conceived and designed the study and prepared the manuscript. JZ, MT, AW, and HLW assisted with the analysis and interpretation of the data. XF, LM, and JL cultivated and obtained leaf samples with different treatments. SY participated in the design of the experiments and provided a critical review. All authors have read, edited, and approved the current version of the manuscript.

## Conflict of Interest

The authors declare that the research was conducted in the absence of any commercial or financial relationships that could be construed as a potential conflict of interest.

## Footnotes

1 https://cottonfgd.org/about/download.html

2 https://www.ncbi.nlm.nih.gov/

3 http://pfam.xfam.org, PF02705

4 https://itol.embl.de/

5 http://gsds.cbi.pku.edu.cn/

6 http://bioinformatics.psb.ugent.be/webtools/plantcare/html/

7 http://www.cottonfgd.org/

## Supplementary Material

Supplementary Table 1 Primers for qPCR.

Supplementary Table 2 Detailed physicochemical characteristics of HAK/KUP/KT proteins of *G. arboreum*, *G. raimondii*, *G. hirsutum*, and *G. barbadense*.

Supplementary Table 3 Characterization of specific *cis*-elements in the promoters of *GhHAK* genes.

Supplementary Table 4 The distribution of *cis*-elements in proximal and distal region of promoter from TSS.
