# Corrigendum: Genome-Wide Identification and Expression Pattern Analysis of the *HAK/KUP/KT* Gene Family of Cotton in Fiber Development and Under Stresses

**DOI:** 10.3389/fgene.2021.632854

**Published:** 2021-07-19

**Authors:** Xu Yang, Jingjing Zhang, Aimin Wu, Hengling Wei, Xiaokang Fu, Miaomiao Tian, Liang Ma, Jianhua Lu, Hantao Wang, Shuxun Yu

**Affiliations:** ^1^School of Agronomy Sciences, Zhengzhou University, Zhengzhou, China; ^2^State Key Laboratory of Cotton Biology, Institute of Cotton Research of CAAS, Anyang, China

**Keywords:** HAK/KUP/KT, cotton, expression patterns, fiber development, stress

In the original article, there were mistakes in Figures 8, 9, and 10 as published. Figures 8 & 10 and their captions were swapped Figure 9 had an incorrect caption. Also one of the Gene name was mislabeled in all the above mentioned figures. GhPOT1 should be GhPOT1-1. The corrected Figures 8, 9, and 10 appear below.

**Figure 8:**
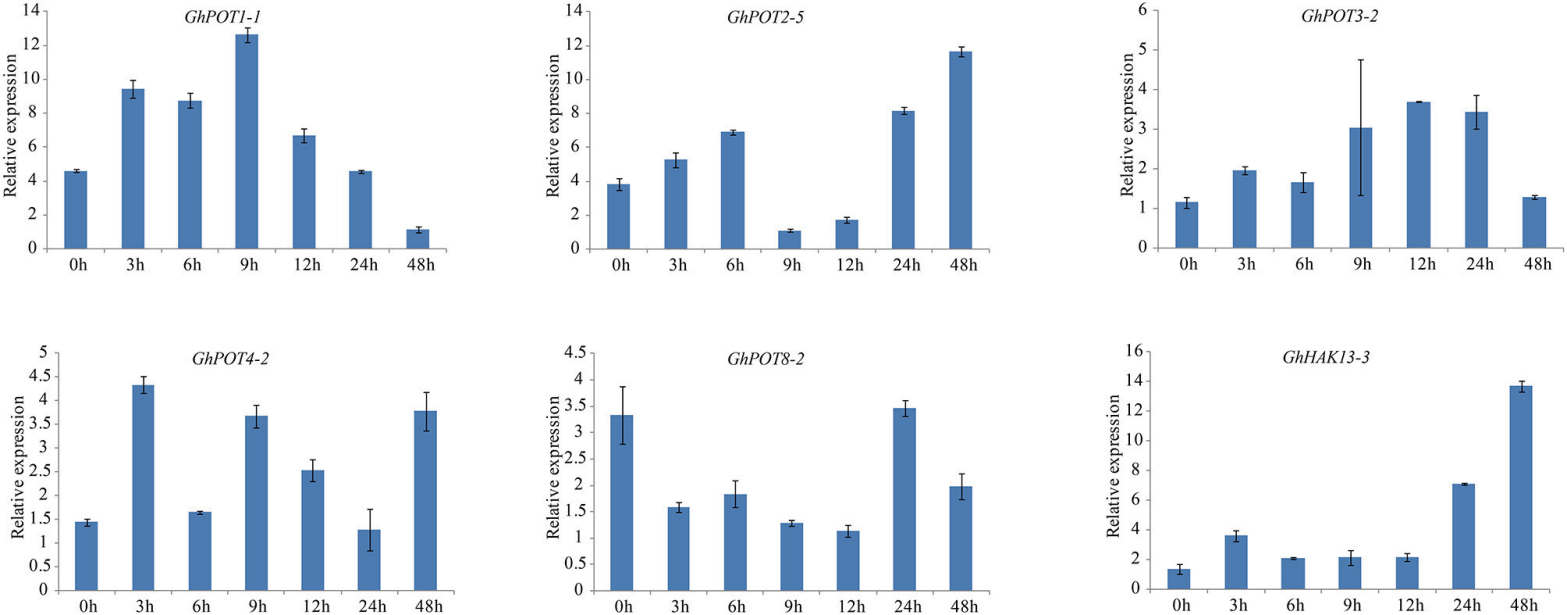
Expressions of GhPOT genes in response to potassium deficiency stress (0.03 mM KCl). The expressions of the GhPOT genes were determined by qRT-PCR using the total RNA isolated from TM-1 leaves at different time points (0, 3, 6, 9, 12, 24 and 48 h) of dehydration stress. The error bars indicate the standard error (SE) of three biological replicates.

**Figure 9:**
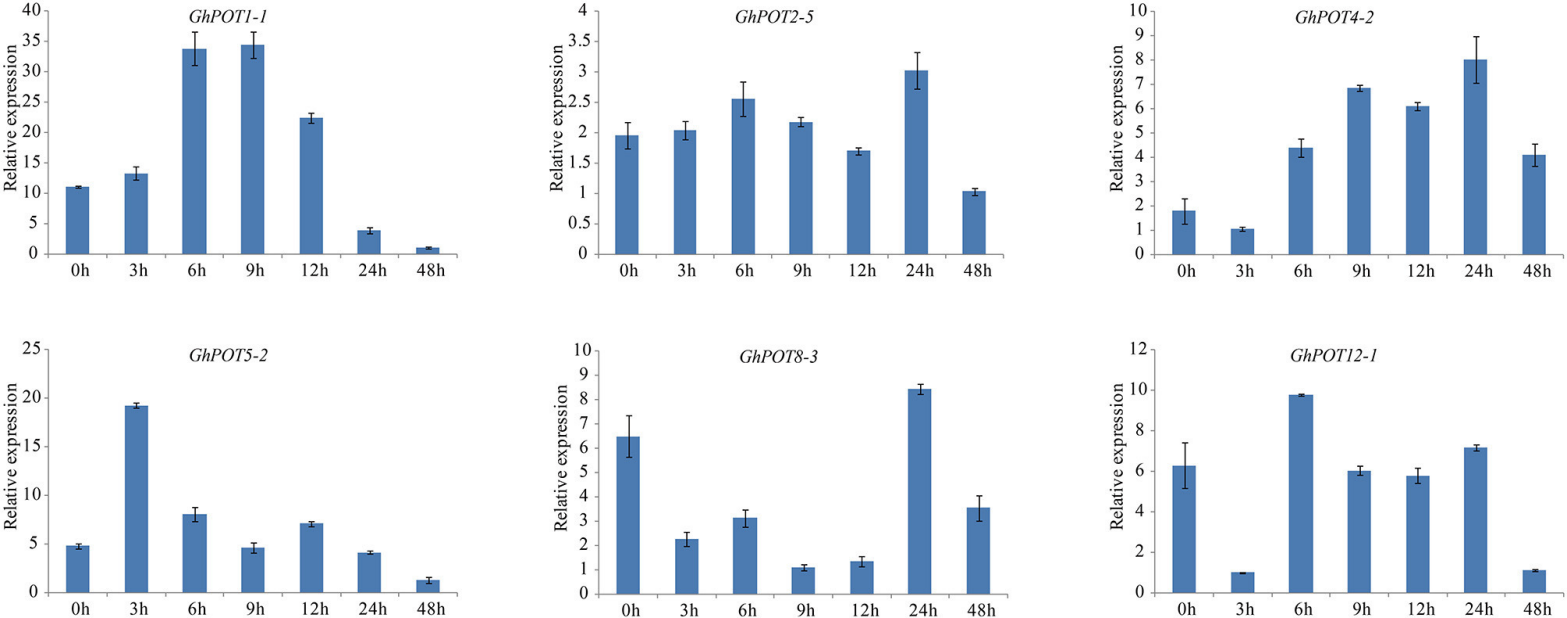
Expressions of GhPOT genes in response to dehydration stress (18% PEG6000). The expressions of GhPOT genes were determined by qRT-PCR using the total RNA isolated from TM-1 leaves at different time points (0, 3, 6, 9, 12, 24 and 48 h) of salt stress. The error bars indicate the standard error (SE) of three biological replicates.

**Figure 10:**
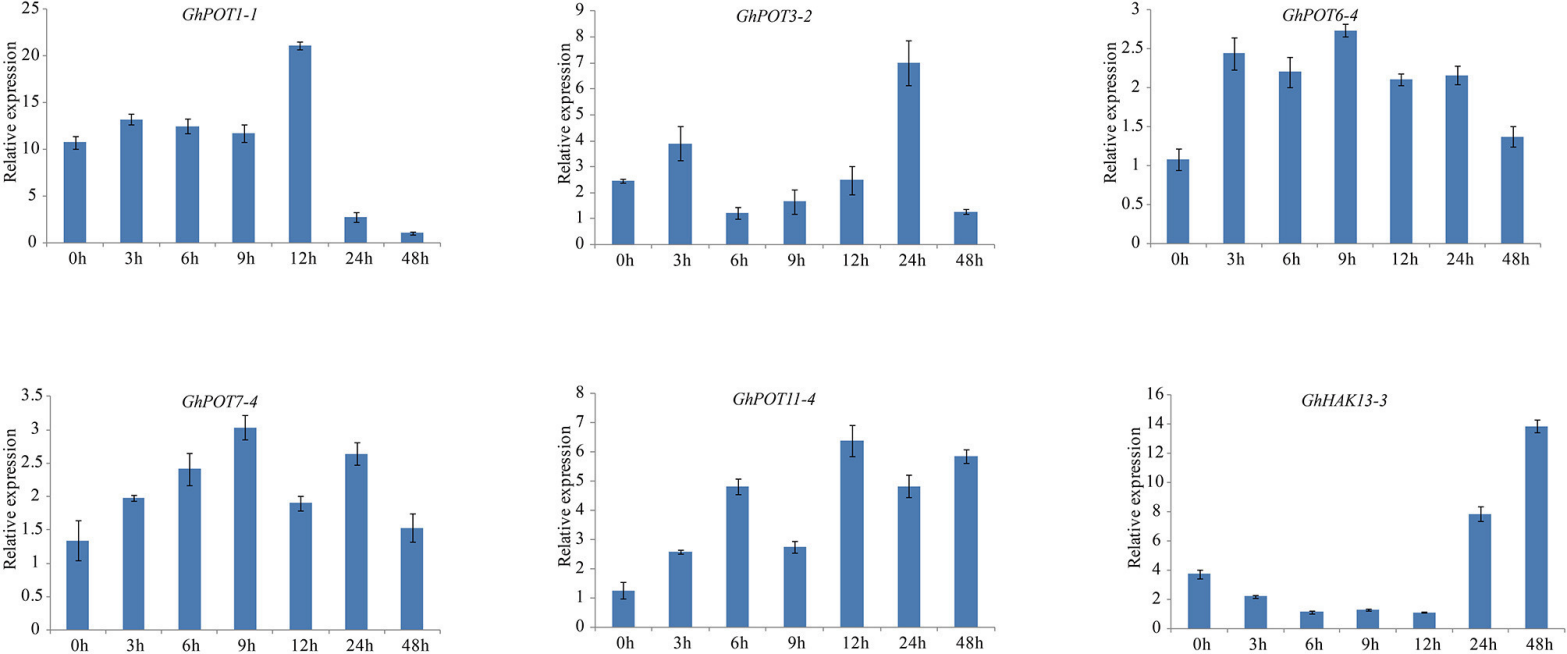
Expressions of GhPOT genes in response to salt stress (300 mM NaCl). The expressions of the GhPOT genes were determined by qRT-PCR using the total RNA isolated from TM-1 leaves at different time points (0, 3, 6, 9, 12, 24 and 48 h) of potassium deficiency stress. The error bars indicate the standard error (SE) of three biological replicates.

Additionally, there were mistakes in the below given sections: In the section: Results and sub-section: qRT-PCR Expression Analysis in Different Fiber Development Stages, the gene name was wrong, *GhPOT12-1* should be as *GHPOT12-2*. The corrected paragraph appears below:

To investigate the possible functions of HAK/KUP/KT in fiber development, we selected 6 genes, which showed high expression levels in different fiber development stages in the transcriptome data (**Figure 5**) to examine their expression patterns. The results (**Figure 7**) showed that different genes had different expression patterns. The expression level of *GhHAK4-3* was high in each period of fiber synthesis (especially 0 DPA ovule), except in 10 DPA fiber. *GhPOT12-2* was significantly expressed in 0 DPA ovules, which is the initiation stage in fiber development. *GhPOT11-3* and *GhHAK13-1* were highly expressed in 5 and 10 DPA fibers, which are the expansion stages in fiber development. *GhPOT2-4* and *GhPOT8-2* were highly expressed in 20 and 25 DPA fibers, which are the secondary wall synthesis stages in fiber development.

In the Section: Results, Sub-section: qRT-PCR Expression Analysis in Response to Multiple Stress Treatments, Paragraph 2, there was an incorrect citation of **Figures 6**, 8, it should be Figures 8, **6**. The corrected paragraph appears below:

As shown in Figures 8, **6**, random selected genes were examined under potassium deficiency stress, and all showed basically the same expression pattern—after potassium deficiency treatment, the expression was upregulated for a few hours and then downregulated. *GhPOT1-1* and *GhPOT3-2* precisely followed this pattern, arriving at their peaks at 9 h and 12 h, respectively. *GhHAK13-3* and *GhPOT2-5*, showed upregulated expressions after the basic pattern and reached their maximum expression levels at 48 h. Although *GhPOT8-2* was downregulated after treatment, the maximum value was reached at 24 h after treatment, and its change trend followed the pattern.

Lastly, in the Section: Discussion, Sub section: Salt Stress, there was an incorrect citation of **Figures 6**, 9, it should be **Figures 6**, 10. The corrected paragraph appears below:

Salt stress is an abiotic stress relevant to modern agricultural production. In this study, transcriptome analysis indicated that most GhPOT genes were responsive to salt stress, and the qRT-PCR results showed that all the selected genes were upregulated after treatment with high concentrations of NaCl. There have been some reports of HAK/KUP/KT genes in other species that could relieve salt stress in plants. Salt stress significantly decreased the root net K^+^ uptake rate in WT rice and almost completely blocked net K^+^ uptake in *Oshak1* mutants when the K^+^ concentration was below 0.05 mm. However, plants overexpressing *OsHAK1* were more tolerant of salt stress compared to the wild type. The same results were shown in *HvHAK1, LeHAK5*, and *CaHAK1* (Martínez-Cordero et al., 2004; Nieves-Cordones et al., 2007; Fulgenzi et al., 2008). *GhPOT5*, which is a homolog of *OsHAK1*, showed higher expression after treatment than did other genes (Figure 6). *AtHKT1* provides a key mechanism for protecting leaves from salt stress (Hamamoto et al., 2015), and *GhPOT1*, which is homologous to *AtHKT1*, showed significantly increased expression after salt treatment (**Figures 6**, 10).

The authors apologize for this error and state that this does not change the scientific conclusions of the article in any way. The original article has been updated.
