# Supplementary material for: Genome-Wide Identification and Expression Pattern Analysis of the HAK/KUP/KT Gene Family of Cotton in Fiber Development and Under Stresses
Source: Front Genet. 2020 Nov 19;11:566469. doi: 10.3389/fgene.2020.566469 (PMC7710864; doi:10.3389/fgene.2020.566469)
Supplement: Supplementary Table 1 — Primers for qPCR. [file Table_1.DOCX]

Table S1 Primers for qPCR

| Gene name | Primer F (5`→3`) | Primer R (5`→3`) |
| --- | --- | --- |
| GhPOT7-4 | AAGAGGTAAACCCCAGCTCCCC | TAAGATGTGAATGCGGCACGCT |
| GhPOT4-2 | TGGACTTGCATGCATTACGGTGA | AAGAGGCACCCATCCTCCTTGA |
| GhPOT11-4 | GTAAGCAGCCAGACGGAGACAG | CCCTCACCACTGTGTTTCCCAG |
| GhHAK13-3 | GGCAATGCATATGGTCTTGCGG | TGCAAGGCATGCAGAGCAGTAA |
| GhPOT6-4 | CGACCCATTTGGACGGGATTCA | CCTAGCTTCCGTAAGTTCCCGC |
| GhPOT12-1 | GGACATATCCACTTGGCGCACT | CAAAGGGCTTGTGCCCATGTCA |
| GhPOT2-5 | GCGTTATCTAGTGGGCCGTGTT | AGTGGGAAGCATCATCTTCGGC |
| GhPOT3-2 | GAGCTAGAGCAGGCCCTTAGGA | AGCAGAGAAAACTTGGCGTGCT |
| GhPOT6-1 | CGGTTTGGCACCACAATCAAGT | CAGACACCGCAGAGAAAACGGA |
| GhPOT1-1 | TCATGCGTCATCATTGTGGGGC | AAGCCATGCTGTGACAATCGGA |
| GhPOT8-2 | AGGATCGGATTCTACGTGTCCGT | TCGGAAACCGATGGTTACAGCC |
| GhPOT8-1 | GCATGAAGATGGTGATGTGGATTCG | CCTCACCTTCTCCGGGGACTTT |
| GhPOT8-4 | TATCGTTCGGGTTCGTGGCATC | TGGACCAGCTCTTCCGACTAGG |
| GhPOT8-3 | GTCGGTCCCAAGGAATACAGGC | CCTTCGCTCTGAAGTGCCAACA |
| GhPOT5-2 | CGGTGAAGGTGGAACATTTGCG | GGCTGGATGGAGTGTCCAACTG |
