# Supplementary material for: Genome-Wide Identification and Expression Pattern Analysis of the HAK/KUP/KT Gene Family of Cotton in Fiber Development and Under Stresses
Source: Front Genet. 2020 Nov 19;11:566469. doi: 10.3389/fgene.2020.566469 (PMC7710864; doi:10.3389/fgene.2020.566469)
Supplement: Supplementary Table 2 — Detailed physicochemical characteristics of HAK/KUP/KT proteins of G. arboreum, G. raimondii, G. hirsutum, and G. barbadense. [file Table_2.DOCX]

Table S2 *HAK/KUP/KT* genes in four cotton species

| Gene ID | Name | Chromosome number | Location | Number of amino acids (aa) | Molecular weight (kDa) | pI | GRAVY | Subcellular localization |
| --- | --- | --- | --- | --- | --- | --- | --- | --- |
|  |  |  |  |  |  |  |  |  |
| Ga01G1612.1 | GaPOT6-1 | Chr01 | 67717483~67723393 | 779 | 87.40 | 7.83 | 0.356 | PM^①^ |
| Ga01G1710.1 | GaPOT3 | Chr01 | 79227940~79232149 | 776 | 87.41 | 9.29 | 0.396 | PM |
| Ga02G1270.1 | GaPOT5-1 | Chr02 | 89981191~89985611 | 766 | 85.82 | 8.34 | 0.333 | PM |
| Ga03G2788.1 | GaPOT11-1 | Chr03 | 135525659~135528757 | 774 | 87.34 | 7.05 | 0.315 | PM |
| Ga04G0059.1 | GaPOT6-2 | Chr04 | 589390~593279 | 777 | 87.33 | 7.33 | 0.298 | PM |
| Ga04G1306.1 | GaPOT4-1 | Chr04 | 78549494~78554493 | 788 | 87.38 | 8.89 | 0.457 | PM |
| Ga04G1520.1 | GaPOT8 | Chr04 | 86968955~86973009 | 777 | 86.91 | 8.71 | 0.354 | PM |
| Ga04G2003.1 | GaPOT2-1 | Chr04 | 96882250~96886888 | 792 | 88.53 | 6.61 | 0.353 | PM |
| Ga04G0192.1 | GaPOT7-1 | Chr04 | 2310335~2315137 | 856 | 95.10 | 5.77 | 0.351 | PM |
| Ga05G3567.1 | GaPOT12 | Chr05 | 70490242~70497777 | 804 | 89.65 | 8.44 | 0.449 | PM |
| Ga05G2970.1 | GaPOT2-2 | Chr05 | 31965699~31970537 | 811 | 90.64 | 7.02 | 0.356 | PM |
| Ga05G3450.1 | GaHAK17 | Chr05 | 53052682~53056788 | 730 | 81.33 | 7.60 | 0.439 | PM |
| Ga08G2359.1 | GaPOT1 | Chr08 | 123213370~123217234 | 755 | 83.96 | 8.29 | 0.433 | PM |
| Ga10G2580.1 | GaHAK13 | Chr10 | 124839846~124844111 | 742 | 82.20 | 8.38 | 0.419 | PM |
| Ga11G2828.1 | GaPOT5-2 | Chr11 | 111709038~111726464 | 719 | 81.44 | 9.20 | 0.370 | PM |
| Ga11G3420.1 | GaPOT4-2 | Chr11 | 118519715~118526696 | 939 | 104.58 | 8.48 | 0.227 | PM |
| Ga11G1031.1 | GaPOT5-3 | Chr11 | 18328963~18331835 | 739 | 83.54 | 8.70 | 0.276 | PM |
| Ga12G0070.1 | GaPOT2-3 | Chr12 | 536174~539694 | 791 | 88.63 | 8.65 | 0.256 | PM |
| Ga12G2632.1 | GaPOT11-2 | Chr12 | 97734054~97738458 | 792 | 88.70 | 8.22 | 0.319 | PM |
| Ga12G2769.1 | GaPOT7-2 | Chr12 | 101041865~101048087 | 858 | 95.19 | 5.14 | 0.337 | PM |
| Ga12G2824.1 | GaPOT5-4 | Chr12 | 101827349~101831627 | 775 | 86.91 | 6.37 | 0.205 | PM |
| Gorai.009G343300.1 | GrPOT12 | Chr09 | 40416842~40423686 | 842 | 93.79 | 6.04 | 0.345 | PM |
| Gorai.009G292800.1 | GrPOT2-1 | Chr09 | 25343869~25349504 | 826 | 92.37 | 6.89 | 0.328 | PM |
| Gorai.004G232600.1 | GrPOT1 | Chr04 | 56942488~56946678 | 755 | 84.13 | 8.01 | 0.424 | PM |
| Gorai.007G129800.1 | GrPOT5-1 | Chr07 | 10421350~10424862 | 760 | 85.58 | 8.99 | 0.272 | PM |
| Gorai.007G281600.1 | GrPOT5-2 | Chr07 | 48266401~48269394 | 757 | 85.44 | 8.77 | 0.288 | PM |
| Gorai.007G071600.1 | GrPOT4-1 | Chr07 | 5028621~5033487 | 619 | 69.19 | 8.54 | 0.514 | PM |
| Gorai.002G170400.1 | GrHAK17 | Chr02 | 42783648~42788079 | 730 | 81.27 | 8.29 | 0.418 | PM |
| Gorai.002G164700.1 | GrPOT6-1 | Chr02 | 39901757~39908411 | 779 | 87.42 | 7.83 | 0.363 | PM |
| Gorai.002G105200.1 | GrHAK13-1 | Chr02 | 13828053~13831908 | 596 | 66.22 | 8.50 | 0.621 | PM |
| Gorai.002G213100.1 | GrPOT5-3 | Chr02 | 56152947~56157078 | 809 | 90.46 | 8.62 | 0.237 | PM |
| Gorai.002G213000.1 | GrPOT5-4 | Chr02 | 56062501~56065882 | 816 | 91.29 | 8.12 | 0.210 | PM |
| Gorai.003G084100.1 | GrPOT3 | Chr03 | 21120242~21124871 | 776 | 87.48 | 9.29 | 0.405 | PM |
| Gorai.005G267900.1 | GrPOT11-1 | Chr05 | 64002422~64007313 | 813 | 91.54 | 8.20 | 0.329 | PM |
| Gorai.011G051700.1 | GrHAK13-2 | Chr11 | 4045704~4050554 | 783 | 87.42 | 8.76 | 0.390 | PM |
| Gorai.008G049100.1 | GrPOT11-2 | Chr08 | 6826681~6831738 | 795 | 89.04 | 8.47 | 0.313 | PM |
| Gorai.008G291400.1 | GrPOT2-2 | Chr08 | 56580671~56584482 | 791 | 88.57 | 8.82 | 0.241 | PM |
| Gorai.008G015600.1 | GrPOT7-1 | Chr08 | 1773721~1780761 | 858 | 95.11 | 5.14 | 0.338 | PM |
| Gorai.008G009900.1 | GrPOT5-5 | Chr08 | 1162478~1166650 | 771 | 86.53 | 6.25 | 0.236 | PM |
| Gene ID | Name | Chromosome number | Location | Number of amino acids (aa) | Molecular weight (kDa) | pI | GRAVY | Subcellular localization |
|  |  |  |  |  |  |  |  |  |
| Gorai.012G082500.1 | GrPOT4-2 | Chr12 | 13565944~13571466 | 788 | 87.52 | 8.96 | 0.449 | PM |
| Gorai.012G142000.1 | GrPOT7-2 | Chr12 | 31547011~31552552 | 856 | 94.97 | 5.73 | 0.342 | PM |
| Gorai.012G171200.1 | GrPOT6-2 | Chr12 | 34062290~34066055 | 681 | 76.19 | 6.36 | 0.417 | PM |
| Gorai.012G060500.1 | GrPOT8-1 | Chr12 | 8472508~8477573 | 778 | 86.97 | 8.82 | 0.347 | PM |
| Gorai.012G014200.1 | GrPOT2-3 | Chr12 | 1585259~1591046 | 792 | 88.59 | 6.90 | 0.365 | PM |
| Gorai.006G029100.1 | GrPOT8-2 | Chr06 | 7468673~7476998 | 764 | 85.48 | 8.56 | 0.321 | PM |
| Ghir_A01G008620.1 | GhHAK13-1 | A01 | 16246924~16250405 | 494 | 54.57 | 7.49 | 0.69 | PM |
| Ghir_A01G013190.1 | GhPOT6-1 | A01 | 63602110~63608668 | 494 | 68.91 | 7.49 | 0.42 | PM |
| Ghir_A01G013640.1 | GhHAK17-1 | A01 | 73496871~73501679 | 494 | 65.62 | 7.49 | 0.49 | PM |
| Ghir_A01G017310.1 | GhPOT5-1 | A01 | 108566129~108569621 | 494 | 93.34 | 7.49 | 0.21 | PM |
| Ghir_A01G017320.1 | GhPOT5-2 | A01 | 108605790~108615696 | 494 | 88.61 | 7.49 | 0.25 | PM |
| Ghir_A02G011180.1 | GhPOT3-1 | A02 | 49639441~49644192 | 494 | 87.36 | 7.49 | 0.40 | PM |
| Ghir_A03G023570.1 | GhPOT11-3 | A03 | 112906924~112909704 | 494 | 74.00 | 7.49 | 0.38 | PM |
| Ghir_A04G012300.1 | GhPOT7-4 | A04 | 80529467~80536306 | 494 | 95.13 | 7.49 | 0.36 | PM |
| Ghir_A04G014720.1 | GhPOT6-4 | A04 | 83798159~83802892 | 494 | 87.36 | 7.49 | 0.30 | PM |
| Ghir_A05G028350.1 | GhPOT2-5 | A05 | 31384206~31390898 | 494 | 88.66 | 7.49 | 0.34 | PM |
| Ghir_A05G033820.1 | GhPOT12-2 | A05 | 73738396~73745516 | 494 | 93.70 | 7.49 | 0.35 | PM |
| Ghir_A05G034540.1 | GhPOT4-3 | A05 | 79450080~79455528 | 494 | 87.42 | 7.49 | 0.46 | PM |
| Ghir_A05G037320.1 | GhPOT8-3 | A05 | 98209027~98214096 | 494 | 86.94 | 7.49 | 0.35 | PM |
| Ghir_A05G041610.1 | GhPOT2-6 | A05 | 107721164~107725684 | 494 | 88.47 | 7.49 | 0.36 | PM |
| Ghir_A09G002750.1 | GhPOT8-4 | A09 | 7969206~7997737 | 494 | 86.74 | 7.49 | 0.33 | PM |
| Ghir_A10G004530.1 | GhHAK13-3 | A10 | 4353500~4358899 | 494 | 82.18 | 7.49 | 0.42 | PM |
| Ghir_A11G006920.1 | GhPOT4-1 | A11 | 6052381~6058538 | 494 | 77.88 | 7.49 | 0.46 | PM |
| Ghir_A11G012390.1 | GhHAK19 | A11 | 12532677~12536046 | 494 | 57.94 | 7.49 | 0.39 | PM |
| Ghir_A11G027350.1 | GhPOT5-3 | A11 | 107708435~107711355 | 494 | 87.09 | 7.49 | 0.27 | PM |
| Ghir_A12G000970.1 | GhPOT5-4 | A12 | 1214864~1219332 | 494 | 80.91 | 7.49 | 0.23 | PM |
| Ghir_A12G001500.1 | GhPOT7-1 | A12 | 1965870~1973582 | 494 | 95.16 | 7.49 | 0.35 | PM |
| Ghir_A12G004840.1 | GhPOT11-1 | A12 | 9285120~9290630 | 494 | 89.03 | 7.49 | 0.32 | PM |
| Ghir_A12G028360.1 | GhPOT2-1 | A12 | 107084667~107088479 | 494 | 86.43 | 7.49 | 0.29 | PM |
| Ghir_D01G014590.1 | GhPOT6-2 | D01 | 38475782~38482440 | 494 | 87.45 | 7.49 | 0.36 | PM |
| Ghir_D01G014890.1 | GhHAK17-2 | D01 | 40822144~40826736 | 494 | 81.19 | 7.49 | 0.42 | PM |
| Ghir_D01G018840.1 | GhPOT5-5 | D01 | 56180398~56183790 | 494 | 91.22 | 7.49 | 0.22 | PM |
| Ghir_D01G018860.1 | GhPOT5-6 | D01 | 56274058~56277908 | 494 | 91.29 | 7.49 | 0.22 | PM |
| Ghir_D02G025020.1 | GhPOT11-2 | D02 | 69667264~69679186 | 494 | 89.63 | 7.49 | 0.32 | PM |
| Ghir_D03G008710.1 | GhPOT3-2 | D03 | 30354096~30358885 | 494 | 87.39 | 7.49 | 0.41 | PM |
| Ghir_D04G001330.1 | GhPOT2-2 | D04 | 1586603~1590481 | 494 | 69.45 | 7.49 | 0.43 | PM |
| Ghir_D04G005710.1 | GhPOT8-1 | D04 | 9232076~9237165 | 494 | 87.01 | 7.49 | 0.35 | PM |
| Ghir_D04G007800.1 | GhPOT4-2 | D04 | 14533075~14538446 | 494 | 86.41 | 7.49 | 0.42 | PM |
| Ghir_D04G016710.1 | GhPOT7-2 | D04 | 51973283~51978855 | 494 | 63.30 | 7.49 | 0.49 | PM |
| Ghir_D04G019420.1 | GhPOT6-3 | D04 | 54845076~54854336 | 494 | 79.54 | 7.49 | 0.38 | PM |
| Ghir_D05G028350.1 | GhPOT2-3 | D05 | 27627724~27634699 | 494 | 88.63 | 7.49 | 0.35 | PM |
| Gene ID | Name | Chromosome number | Location | Number of amino acids (aa) | Molecular weight (kDa) | pI | GRAVY | Subcellular localization |
|  |  |  |  |  |  |  |  |  |
| Ghir_D05G032990.1 | GhPOT12-1 | D05 | 44175845~44183108 | 494 | 93.79 | 7.49 | 0.35 | PM |
| Ghir_D08G021850.1 | GhPOT1-1 | D08 | 63232760~63236948 | 494 | 65.59 | 7.49 | 0.54 | PM |
| Ghir_D09G002720.1 | GhPOT8-2 | D09 | 8118893~8127306 | 494 | 85.54 | 7.49 | 0.32 | PM |
| Ghir_D10G005320.1 | GhHAK13-2 | D10 | 4646051~4652032 | 494 | 64.54 | 7.49 | 0.48 | PM |
| Ghir_D11G012370.1 | GhPOT5-7 | D11 | 11300262~11303831 | 494 | 85.69 | 7.49 | 0.26 | PM |
| Ghir_D11G027470.1 | GhPOT5-8 | D11 | 58712302~58715220 | 494 | 87.12 | 7.49 | 0.27 | PM |
| Ghir_D12G000980.1 | GhPOT5-9 | D12 | 1173708~1178139 | 494 | 86.48 | 7.49 | 0.23 | PM |
| Ghir_D12G001530.1 | GhPOT7-3 | D12 | 1827822~1834886 | 494 | 95.14 | 7.49 | 0.34 | PM |
| Ghir_D12G004810.1 | GhPOT11-4 | D12 | 7147629~7152998 | 494 | 64.95 | 7.49 | 0.52 | PM |
| Ghir_D12G028500.1 | GhPOT2-4 | D12 | 62078612~62083210 | 494 | 85.19 | 7.49 | 0.22 | PM |
| Gbar_A01G008710.1 | GbHAK13-1 | A01 | 15640546~15644013 | 696 | 77.69 | 8.77 | 0.452 | PM |
| Gbar_A01G013520.1 | GbPOT6-1 | A01 | 60198002~60203912 | 763 | 85.68 | 8.03 | 0.369 | PM |
| Gbar_A01G014020.1 | GbHAK17-1 | A01 | 71731101~71735817 | 730 | 81.32 | 8.12 | 0.429 | PM |
| Gbar_A01G017800.1 | GbPOT5-1 | A01 | 106664484~106667692 | 809 | 90.68 | 8.56 | 0.227 | PM |
| Gbar_A01G017820.1 | GbPOT5-2 | A01 | 106704202~106714004 | 790 | 88.61 | 8.48 | 0.245 | PM |
| Gbar_A02G010990.1 | GbPOT3-1 | A02 | 47164263~47169090 | 758 | 85.27 | 9.35 | 0.385 | PM |
| Gbar_A11G006490.1 | GbPOT4-1 | A11 | 5653954~5659066 | 786 | 87.68 | 8.06 | 0.448 | PM |
| Gbar_A11G026700.1 | GbHAK1 | A11 | 96869348~96872704 | 623 | 70.80 | 8.04 | 0.265 | PM |
| Gbar_A12G000890.1 | GbPOT5-3 | A12 | 1081130~1085389 | 775 | 86.97 | 6.49 | 0.201 | PM |
| Gbar_A12G001410.1 | GbPOT7-1 | A12 | 1806695~1813707 | 858 | 95.19 | 5.18 | 0.342 | PM |
| Gbar_A12G004820.1 | GbPOT11-1 | A12 | 9320966~9326534 | 795 | 89.00 | 8.24 | 0.312 | PM |
| Gbar_A12G028280.1 | GbPOT2-1 | A12 | 101568597~101574232 | 772 | 86.38 | 8.59 | 0.289 | PM |
| Gbar_D01G014640.1 | GbPOT6-2 | D01 | 39047252~39053822 | 779 | 87.44 | 7.83 | 0.357 | PM |
| Gbar_D01G014900.1 | GbHAK17-2 | D01 | 41295587~41300127 | 651 | 72.80 | 8.75 | 0.447 | PM |
| Gbar_D01G018950.1 | GbPOT5-4 | D01 | 56164790~56168143 | 816 | 91.23 | 8.15 | 0.220 | PM |
| Gbar_D01G018970.1 | GbPOT5-5 | D01 | 56254677~56259017 | 816 | 91.35 | 8.67 | 0.216 | PM |
| Gbar_D02G025540.1 | GbPOT11-2 | D02 | 67543124~67548889 | 789 | 88.57 | 8.85 | 0.370 | PM |
| Gbar_D03G008170.1 | GbPOT3-2 | D03 | 29347554~29352147 | 777 | 87.57 | 9.24 | 0.418 | PM |
| Gbar_D04G001260.1 | GbPOT2-2 | D04 | 1489264~1493904 | 792 | 88.51 | 6.70 | 0.364 | PM |
| Gbar_D04G005690.1 | GbPOT8-1 | D04 | 8710712~8715761 | 615 | 69.25 | 8.97 | 0.386 | PM |
| Gbar_D04G007750.1 | GbPOT4-2 | D04 | 13988551~13993896 | 698 | 77.64 | 9.14 | 0.455 | PM |
| Gbar_D04G016880.1 | GbPOT7-2 | D04 | 48119732~48125232 | 826 | 91.43 | 5.47 | 0.329 | PM |
| Gbar_D04G019290.1 | GbPOT6-3 | D04 | 51026028~51035147 | 694 | 78.05 | 5.78 | 0.393 | PM |
| Gbar_D05G028210.1 | GbPOT2-3 | D05 | 27346989~27353640 | 796 | 88.54 | 6.75 | 0.338 | PM |
| Gbar_A03G023730.1 | GbPOT11-3 | A03 | 105233087~105235861 | 671 | 75.18 | 6.98 | 0.371 | PM |
| Gbar_D08G022430.1 | GbPOT1-1 | D08 | 60369471~60372302 | 590 | 65.65 | 8.74 | 0.544 | PM |
| Gbar_D09G002750.1 | GbPOT8-2 | D09 | 8206817~8215062 | 764 | 85.58 | 8.56 | 0.323 | PM |
| Gbar_D11G012680.1 | GbPOT5-6 | D11 | 11062270~11065889 | 760 | 85.65 | 8.99 | 0.286 | PM |
| Gbar_D11G027670.1 | GbPOT5-7 | D11 | 55892460~55895065 | 675 | 76.18 | 8.74 | 0.310 | PM |
| Gbar_D12G001030.1 | GbPOT5-8 | D12 | 1177192~1181446 | 771 | 86.43 | 6.44 | 0.229 | PM |
| Gbar_A12G001410.1 | GbPOT7-3 | A12 | 1806695~1813707 | 858 | 95.14 | 5.14 | 0.337 | PM |
| Gene ID | Name | Chromosome number | Location | Number of amino acids (aa) | Molecular weight (kDa) | pI | GRAVY | Subcellular localization |
|  |  |  |  |  |  |  |  |  |
| Gbar_D12G004830.1 | GbPOT11-4 | D12 | 6895560~6901049 | 795 | 89.07 | 8.47 | 0.312 | PM |
| Gbar_D12G028200.1 | GbPOT6-4 | D12 | 58492642~58497579 | 584 | 65.13 | 9.11 | 0.448 | PM |
| Gbar_A04G012200.1 | GbPOT7-4 | A04 | 77349509~77355064 | 811 | 90.25 | 6.64 | 0.420 | PM |
| Gbar_A04G014560.1 | GbPOT6-5 | A04 | 80192823~80196970 | 777 | 87.38 | 7.32 | 0.270 | PM |
| Gbar_A05G027380.1 | GbPOT2-4 | A05 | 30073626~30080320 | 803 | 89.44 | 6.78 | 0.350 | PM |
| Gbar_A05G032780.1 | GbPOT12-2 | A05 | 69190635~69197542 | 842 | 93.83 | 6.04 | 0.346 | PM |
| Gbar_A05G033500.1 | GbPOT4-3 | A05 | 74388519~74393974 | 787 | 87.35 | 8.90 | 0.458 | PM |
| Gbar_A05G036520.1 | GbPOT8-3 | A05 | 92058772~92063821 | 777 | 86.92 | 8.77 | 0.354 | PM |
| Gbar_A05G040660.1 | GbPOT2-5 | A05 | 101070264~101074904 | 792 | 88.47 | 6.54 | 0.362 | PM |
| Gbar_A08G021670.1 | GbPOT1-2 | A08 | 113823939~113828004 | 755 | 83.99 | 8.39 | 0.431 | PM |
| Gbar_A09G002920.1 | GbPOT8-4 | A09 | 7755742~7784306 | 772 | 86.68 | 8.75 | 0.326 | PM |
| Gbar_A10G005180.1 | GbHAK13-2 | A10 | 4650337~4655862 | 742 | 82.20 | 8.29 | 0.420 | PM |
| Gbar_D10G027520.1 | GbHAK13-3 | Scaffold3708 | 16445~21932 | 783 | 87.42 | 8.76 | 0.390 | PM |
| ①:Plasma Membrane |  |  |  |  |  |  |  |  |
