# Supplementary material for: Genome-Wide Identification and Expression Pattern Analysis of the HAK/KUP/KT Gene Family of Cotton in Fiber Development and Under Stresses
Source: Front Genet. 2020 Nov 19;11:566469. doi: 10.3389/fgene.2020.566469 (PMC7710864; doi:10.3389/fgene.2020.566469)
Supplement: Supplementary Table 3 — Characterization of specific cis-elements in the promoters of GhHAK genes. [file Table_3.DOCX]

Table S3 Characterization of specific cis-elements in the promoters of *GhHAK* genes

| Site name | Sequence | Function | No. of *GhPOTs* |
| --- | --- | --- | --- |
| circadian | CAAAGATATC | circadian control | 11 |
| RY-element | CATGCATG | seed-specific regulation | 4 |
| CAT-box | GCCACT | meristem expression | 12 |
| GCN4_motif | TGAGTCA | endosperm expression | 6 |
| AACA_motif | TAACAAACTCCA | endosperm-specific negative regulation | 5 |
| MSA-like | CAACGGA | cell cycle regulation | 1 |
