# Supplementary material for: Genome-Wide Identification and Expression Pattern Analysis of the HAK/KUP/KT Gene Family of Cotton in Fiber Development and Under Stresses
Source: Front Genet. 2020 Nov 19;11:566469. doi: 10.3389/fgene.2020.566469 (PMC7710864; doi:10.3389/fgene.2020.566469)
Supplement: Supplementary Table 4 — The distribution of cis-elements in proximal and distal region of promoter from TSS. [file Table_4.DOCX]

Table S4 the distribution of cis-elements in  proximal and distal region of promoter from TSS

| Name | Location | MYB | ABRE | DRE | LTR | MBS | TC-rich |
| --- | --- | --- | --- | --- | --- | --- | --- |
| GhHAK13-1 | 1bp~1000bp | 2 | 1 |  |  |  |  |
|  | 1001bp~2000bp | 2 | 1 |  |  |  | 1 |
| GhPOT6-1 | 1bp~1000bp | 1 |  |  |  |  |  |
|  | 1001bp~2000bp |  |  |  |  |  | 1 |
| GhHAK17-1 | 1bp~1000bp | 1 | 1 |  |  |  |  |
|  | 1001bp~2000bp | 1 | 1 |  |  |  | 2 |
| GhPOT5-1 | 1bp~1000bp | 3 | 4 |  |  | 1 | 1 |
|  | 1001bp~2000bp |  |  |  |  |  |  |
| GhPOT5-2 | 1bp~1000bp | 5 | 3 |  |  |  |  |
|  | 1001bp~2000bp | 5 | 3 |  |  | 1 |  |
| GhPOT3-1 | 1bp~1000bp | 5 | 2 |  | 2 | 1 |  |
|  | 1001bp~2000bp |  |  | 1 |  |  |  |
| GhPOT11-3 | 1bp~1000bp |  |  |  |  |  |  |
|  | 1001bp~2000bp | 1 |  | 1 |  | 1 |  |
| GhPOT7-4 | 1bp~1000bp |  |  |  |  |  | 1 |
|  | 1001bp~2000bp | 1 | 1 |  |  |  |  |
| GhPOT6-4 | 1bp~1000bp |  | 1 |  |  |  |  |
|  | 1001bp~2000bp | 2 |  |  |  | 1 |  |
| GhPOT2-5 | 1bp~1000bp |  | 2 |  |  |  |  |
|  | 1001bp~2000bp | 6 |  |  |  | 1 | 1 |
| GhPOT12-2 | 1bp~1000bp | 3 | 4 |  |  |  |  |
|  | 1001bp~2000bp | 3 |  |  |  |  |  |
| GhPOT4-3 | 1bp~1000bp | 1 |  |  | 1 |  |  |
|  | 1001bp~2000bp | 1 |  |  |  |  |  |
| GhPOT8-3 | 1bp~1000bp | 2 |  |  | 1 |  |  |
|  | 1001bp~2000bp | 2 |  |  |  |  | 1 |
| GhPOT2-6 | 1bp~1000bp | 1 | 1 |  |  |  | 1 |
|  | 1001bp~2000bp | 2 | 2 |  |  |  |  |
| GhPOT8-4 | 1bp~1000bp | 1 | 3 |  |  |  |  |
|  | 1001bp~2000bp | 1 |  |  |  |  | 1 |
| GhHAK13-3 | 1bp~1000bp |  |  |  | 1 |  | 1 |
|  | 1001bp~2000bp |  |  |  |  |  |  |
| GhPOT4-1 | 1bp~1000bp | 3 | 1 |  |  | 1 | 1 |
|  | 1001bp~2000bp | 1 | 3 |  |  | 1 |  |
| GhHAK19 | 1bp~1000bp |  |  |  |  |  | 1 |
|  | 1001bp~2000bp |  |  |  |  |  |  |
| GhPOT5-3 | 1bp~1000bp | 3 | 2 |  |  | 1 |  |
|  | 1001bp~2000bp | 3 |  |  |  |  |  |
| GhPOT5-4 | 1bp~1000bp | 3 | 1 |  |  |  |  |
|  | 1001bp~2000bp | 3 | 2 |  | 1 | 1 |  |
| GhPOT7-1 | 1bp~1000bp | 2 | 4 |  | 1 |  |  |
|  | 1001bp~2000bp |  | 1 |  |  |  | 1 |
| GhPOT11-1 | 1bp~1000bp | 1 | 3 |  |  |  |  |
|  | 1001bp~2000bp | 4 | 1 | 1 |  |  |  |
| GhPOT2-1 | 1bp~1000bp | 1 | 1 |  |  |  |  |
|  | 1001bp~2000bp | 4 | 1 | 1 | 1 | 2 |  |
| GhPOT6-2 | 1bp~1000bp | 3 |  |  |  |  |  |
|  | 1001bp~2000bp | 3 |  |  |  | 1 | 1 |
| GhHAK17-2 | 1bp~1000bp | 5 |  |  |  | 2 |  |
|  | 1001bp~2000bp | 1 |  |  |  | 1 |  |
| GhPOT5-5 | 1bp~1000bp | 1 | 1 |  |  | 1 |  |
|  | 1001bp~2000bp | 1 |  |  |  |  |  |
| GhPOT5-6 | 1bp~1000bp | 7 |  |  |  |  |  |
|  | 1001bp~2000bp | 8 |  |  |  | 1 |  |
| GhPOT11-2 | 1bp~1000bp | 6 | 3 |  |  |  | 1 |
|  | 1001bp~2000bp | 3 |  |  |  |  |  |
| GhPOT3-2 | 1bp~1000bp | 5 | 3 |  | 1 | 2 |  |
|  | 1001bp~2000bp |  |  | 1 | 1 |  |  |
| GhPOT2-2 | 1bp~1000bp | 3 | 2 |  |  |  |  |
|  | 1001bp~2000bp | 6 |  | 1 | 1 | 2 |  |
| GhPOT8-1 | 1bp~1000bp | 2 | 3 | 1 |  |  |  |
|  | 1001bp~2000bp | 2 |  |  |  |  | 1 |
| GhPOT4-2 | 1bp~1000bp |  |  |  |  |  |  |
|  | 1001bp~2000bp | 2 |  |  |  |  |  |
| GhPOT7-2 | 1bp~1000bp | 3 | 1 | 1 |  |  |  |
|  | 1001bp~2000bp | 5 |  |  |  | 1 |  |
| GhPOT6-3 | 1bp~1000bp | 2 |  |  |  | 1 |  |
|  | 1001bp~2000bp | 1 | 1 |  |  | 1 |  |
| GhPOT2-3 | 1bp~1000bp |  | 2 |  |  |  |  |
|  | 1001bp~2000bp | 6 |  |  |  | 1 | 1 |
| GhPOT12-1 | 1bp~1000bp |  |  | 1 | 1 |  |  |
|  | 1001bp~2000bp | 3 |  |  |  |  |  |
| GhPOT1 | 1bp~1000bp | 8 |  | 1 |  |  |  |
|  | 1001bp~2000bp |  |  |  |  |  |  |
| GhPOT8-2 | 1bp~1000bp | 8 |  |  |  | 1 |  |
|  | 1001bp~2000bp |  |  |  |  |  | 1 |
| GhHAK13-2 | 1bp~1000bp | 1 |  |  |  |  |  |
|  | 1001bp~2000bp |  | 2 |  | 1 |  |  |
| GhPOT5-7 | 1bp~1000bp |  | 1 |  |  |  |  |
|  | 1001bp~2000bp |  | 2 |  |  |  |  |
| GhPOT5-8 | 1bp~1000bp | 1 |  |  |  | 1 |  |
|  | 1001bp~2000bp | 3 | 1 |  |  | 1 |  |
| GhPOT5-9 | 1bp~1000bp | 2 | 2 |  |  |  |  |
|  | 1001bp~2000bp |  |  |  |  |  |  |
| GhPOT7-3 | 1bp~1000bp | 6 | 4 |  |  |  |  |
|  | 1001bp~2000bp |  | 1 |  |  |  | 1 |
| GhPOT11-4 | 1bp~1000bp | 9 |  |  |  |  |  |
|  | 1001bp~2000bp | 2 |  |  |  | 1 |  |
| GhPOT2-4 | 1bp~1000bp | 4 |  |  |  |  |  |
|  | 1001bp~2000bp | 5 |  |  |  | 1 |  |
